# Development and validation of a Screening Questionnaire of Family Mistreatment against Older Adults for use in primary care settings in Mexico

**DOI:** 10.1111/hsc.12466

**Published:** 2017-07-06

**Authors:** María Guadalupe Ruelas-González, Blanca Estela Pelcastre-Villafuerte, Eric Monterrubio-Flores, Jacqueline Elizabeth Alcalde-Rabanal, Doris V. Ortega-Altamirano, Ana Lorena Ruano, Pedro J. Saturno Hernández

**Affiliations:** 1 Centre for Evaluation Research and Survey, National Institute of Public Health, Cuernavaca, México; 2 Centre for Health Systems Research, National Institute of Public Health, Cuernavaca, México; 3 Centre for Nutrition and Health Research, National Institute of Public Health, Cuernavaca, México; 4 Centre for International Health, University of Bergen, Bergen, Norway

**Keywords:** elder abuse, family members, Mexico, mistreatment, screening, validity

## Abstract

The abuse of older adults is a serious public health issue that can be difficult to identify at the first level of care. Medical and nursing personnel are sometimes unable to identify older adults who suffer family mistreatment. This can occur when victims feel shame or as a result of cultural factors. In the light of this, healthcare personnel require a screening tool that can be used to identify signs of mistreatment. The aim of this study was to develop and validate a screening tool for detecting the familial mistreatment of older adults in primary care settings. A mixed method cross-sectional study was carried out in three phases between 2009 and 2012 in Mexico. The formative phase involved using a qualitative methodology to identify terms that older adults use to identify practices defined as forms of mistreatment. On this basis, the second phase involved the design of a screening tool through the formation of items in collaboration with a panel of experts. These items were tested on older adults to ensure their intelligibility. Finally, validity and reliability levels were evaluated through the application of the screening tool to a sample of older adults at a primary care facility and at a legal centre. These findings were discussed with gerontologists, and the data were analysed through an exploratory factor analysis with orthogonal rotation and Cronbach’s alpha using STATA v13. From the results, we generated a screening tool that is culturally and socially tailored to older adults in Mexico. The tool has a Cronbach’s alpha of 0.89, a sensitivity value of 86% (*p* < .05) and a specificity value of 90% (*p* < .05) for positive answers to the tool’s 15 items. Applying this tool at the first level of care could limit damage to older adults’ health and could lower the frequency of emergency room use in hospitals.

## 1 | INTRODUCTION

Today, the world’s population continues to grow older, and as more people reach the age of 60, the number of reports of the mistreatment of older adults continues to increase. Long considered a public health problem, mistreatment is difficult to detect and in many cases goes unreported (Bond & Butler, 2013; Burnett, Achenbaum, & Murphy, 2014; Cooper, Selwood, & Livingston, 2008; WHO 2015; WHO 2002). According to the World Health Organization (WHO), older adult mistreatment is “a single or repeated act, or lack of appropriate action, occurring within any relationship where there is an expectation of trust which causes harm or distress to an older person” (WHO 2002). Such mistreatment can occur within an institution, but it is also commonly performed by a victim’s family members (Adams, 2012; Ruelas-González, Pelcastre-Villafuerte, & Reyes-Morales, 2014).

The prevalence of older adult mistreatment varies greatly across Latin America, and while Costa Rica reports the lowest levels at 3%, such behaviour can be more than 10 times more prevalent in other countries such as Mexico (35.3%) and Chile (37.6%) (Cooper et al., 2008; Huenchuán & Rodríguez-Piñero, 2014; Martínez & Brenes, 2007; Ruelas-González et al., 2016). Latin American countries tend to lack policies for systematic data collection at the health system level, and many of the region’s health systems also lack the capacity to collect and compute such data (LoFaso & Rosen, 2014). In addition, there is little consensus on how to detect older adult mistreatment and on what tools care providers in health, social and legal services should use for this purpose (Almogue, Weiss, Marcus, & Beloosesky, 2009; Bonnie & Wallace, 2003; Krug, Dahlberg, Mercy, Zwi, & Lozano, 2002).

There has been a push to develop valid and reliable data collection tools for the detection of cases of older adult mistreatment for over 30 years, but cultural and socioeconomic barriers remain (Kosberg, Lowenstein, Garcia, & Biggs, 2003; Patterson & Malley-Morrisona, 2006; Podnieks, Penhale, Goergen, Biggs, & Han, 2010). A review of existing data collection tools shows that many of such tools can only be used by specific health professionals while others are designed to be used within larger demographic health surveys. A third category of these tools includes those used through social and legal services (Acierno, Hernandez-Tejada, Muzzy, & Steve, 2009; Aravanis et al., 1993; Bond & Butler, 2013; Hamid et al., 2013; Hwalek & Sengstock, 1985; Hwalek, Sengstock, & Lawrence, 1984; Lindenbach, Larocque, Lavoie, & Garceau, 2012; Sellas, 2004; WHO 2008). In 2008, the WHO presented a tool that could be used at the first level of care (Perel-Levin, 2008), and a tool developed by Burnett et al. (2014) has proven useful for working with older adults with disabilities. However, these tools were developed without considering either the perceptions of older adult about the mistreatment, or the perceptions of personnel who may work in health and other social services (Perel-Levin, 2008; WHO and International Network for the Prevention of Elder Abuse, WHO/INPEA, 2002); such tools also fail to consider the perceptions legal service personnel.

Our review also reveals considerable levels of variability in the formats and time lengths related to tools available today: some involve as little as 5 items while others involve as many as 42, which can translate into significant deterrents for health personnel who are already pressed for time. In addition, we found that most tools address three or more different forms of mistreatment (Bonnie & Wallace, 2003; WHO 2008). The most common ones are physical, emotional, sexual and financial. Only two of the tools can be self-administered while the others must be employed by specially trained personnel. The Geriatric Mistreatment Scale was developed for the Mexican context, but using it requires special training. Two of these tools have a reported sensitivity of greater than 0.70 and only one achieves a specificity level of 0.44. Other psychometric measures reported included construct, content and predictive validity (Bond & Butler, 2013; Burnett et al., 2014). The different measures reported by the tools show that we lack a universal definition of mistreatment and of mistreatment towards older adults, pointing to the difficulties inherent to this task (Perel-Levin, 2008). In relation to this context, our goal was to develop and validate a screening tool for identifying the family mistreatment of older adults in Mexico that can be administered at the first level of care and which is based on the perceptions of older adults, health service providers and legal service personnel.

## 2 | METHODS

A mixed methods cross-sectional study was carried out between June 2009 and February 2012. It was implemented in three phases and involved active participation from older adults and health services personnel at the first and third levels of care. Social institution and legal aid personnel were also involved. To increase the cultural diversity of the study participants, participants were recruited from three cities in Mexico presenting low, moderate and high levels of economic marginalisation in northern, central and southern areas of the country respectively. All of the sites include approximately the same proportion of older adults as the national average of 8.37% (Consejo Nacional de Población 2011).

In each city, two of the largest hospitals were visited to identify the two health centres at the first level of care that had reported the highest frequencies of older adult referrals for any cause over the last 6 months prior to the onset of data collection. In addition, two neighbourhoods positioned adjacent to each of the health centres with the largest number of older adult residents were selected.

### 2.1 | Phase 1: Formative research

The goal of this first phase was to gather the perceptions of older adults and professionals who work with them on family member mistreatment of older adults. A qualitative design was applied, with semi-structured interviews used as our main data collection technique. Through these interviews, we collected nuanced data that allowed us to capture variations in the use of language around older adult mistreatment (Flick, 2007; Rinaldi-Carpenter, 2011). The semi-structured interviews were carried out following interview guides developed specifically for each type of participant: older adults, healthcare providers and social and legal service personnel. The interview questions explored perceptions around mistreatment in general, related causes, everyday situations in which it is possible to identify mistreatment, victims of mistreatment, perpetrators, consequences of mistreatment, actions that could be viewed as forms of older adult mistreatment, manifestations of mistreatment, ways to identify mistreatment and forms of attention required.

At this stage, 163 participants in the three cities were interviewed. Of these, 63 were older adults gathered in the selected neighbourhoods and 100 participants were health, social and legal personnel employed at public institutions located in the same cities.

In total, 63 semi-structured interviews were carried out with older adults (≥60 years of age) who did not present hearing or speech problems and who did not present any signs of cognitive impairment. Before selecting each interviewee, a home visit was carried out to verify that our criteria were being met. To screen for cognitive impairments, we administered Folstein, Folstein, and McHugh’s (1975) mini mental test. We only interviewed older adults who scored 24 or higher on the scale. In addition, 100 semi-structured interviews were carried out with health and social/legal service personnel in their places of work and during work hours. The goal of this approach was to be as unobtrusive as possible. The sample includes physicians (*n* = 40), nurses (*n* = 36) and social workers (*n* = 12) as well as specific personnel who provide care to older adults and in an emergency room. We also interviewed medical advisors available through legal services, lawyers, social workers and psychologists (*n* = 12).

All of the interviews were digitally recorded and transcribed verbatim. The transcripts were organised following a qualitative content analysis, which allowed for the identification of patterns in the interview data (Cresswell, 2007). The texts were independently coded by MGR & BP who then compared and discussed their code lists to reach a consensus based on the thematic categories of interest. The data were processed using the Atlas-ti 6.1 software program (Rinaldi-Carpenter, 2011).

### 2.2 | Phase 2: Tool development

For this phase, analysed data collected from the semi-structured interviews were used to construct a tool that can screen for instances of older adult mistreatment. The research team developed the tool through a series of steps. First, items were developed based on the interviews with older adults and health, legal and social service personnel. The goal was to develop a tool that would take between 10 and 15 min to use with items that employ clear and understandable language that the older adults feel comfortable hearing and using (and that include terms used by older adults for identifying different forms of mistreatment). Finally, each item was to be designed as a closed question requiring a “yes/no” answer (Echeverría, Sotelo, Barrera, & López, 2013).

A panel of experts in topics around providing care to older adults was convened with 17 identified people who had experience in identifying the mistreatment of older adults and who had worked with older adults for at least 2 years in health (*n* = 7), social (*n* = 5) or legal service contexts (*n* = 5) (Barraza-Macías, 2007). The panel provided advice on phrasing and selected items with the greatest capacity to reveal older adult mistreatment. Once the items were discussed, the panel worked independently to assess the relevance of each item and scored each item on a scale of 1–10. A score of 1 was the value given for items considered to be “irrelevant” and a score of 10 was given for items considered to be “very relevant.” Items without a median score of 8 or above were excluded from the tool.

The next step of this phase involved exploring how the items would be understood by the target population of older adults. A total of 10 participants from one health service in each city were selected for a total of 30 purposively sampled older adults who had not participated in the formative phase. Each participant of this step was asked to identify difficult to understand terms. After discussing this with them, the older adults were asked to rephrase each item using their own words. Finally, items were changed to reflect words and terms used by the older adults.

### 2.3 | Phase 3: Screening tool validation

The aim of the third phase was to evaluate the construct and concurrent validity of the tool. To do this, we selected a sample of older adults using feasibility criteria: ten adults for each item found through the tool plus 10% to account for non-responses, creating a total 253 older adults of at least 60 years of age (Holland & Wainer, 2009). Participants who had experienced mistreatment and those that had not were represented in our sample. Older adults who did not present signs of mistreatment were selected based on clinical records and based on reports of not exhibiting signs of physical mistreatment upon arrival at consultations or appointments. Potential participants were invited to participate in this stage of the study by their general practitioners, who administered the tool in a private area available through a health service. Older adults who had experienced mistreatment must have filed at least one complaint of such mistreatment with a legal body. Once identified, a gerontologist invited these individuals to participate and applied the tool in a private area. All of the participants then received a private consultation with a gerontologist/geriatric specialist who evaluated and diagnosed each person as “presenting mistreatment” or as “not presenting mistreatment” using standards presented in the clinical guide for detecting family mistreatment in older adults (Secretaría de Salud 2009). The same procedure was applied in all three cities.

#### 2.3.1 | Construct validity

To evaluate the internal consistency of each item and how each operates as a screening tool of older adult mistreatment, all answers given by the participants were reclassified. Each item was given one point as a valuation dependent on positive responses to mistreatment questions and a value of zero when mistreatment was not present. Following this, through an initial exploratory analysis, for each item, the proportional difference in positive and negative responses to mistreatment was determined via chi-squared tests.

To measure the items’ internal validity as a whole, the degree to which the items capture the “mistreatment of older adults” was estimated through an exploratory factorial analysis (EFA) with orthogonal rotation, which was identified as the best model for this construct (Hair, Black, Babin, & Anderson, 2014; Williams, Onsman, & Brown, 2010). The final model included items with factorial loads of equal to or higher than 0.4 and a Cronbach’s alpha of greater than 0.8 (Quero-Virla, 2010; Santos, 1999).

We constructed a total score (TS) calculated from the sum of all positive (value = 1) and negative answers on mistreatment (value = 0) where values of or close to zero denote a lower probability of mistreatment and where higher values (maximum value = 15) denote a higher probability of mistreatment.

To evaluate the consistency of the items based on the TS, items that confirmed the validity of the construct were used. This was done by estimating the differences in medians for each TS of each item. The observed median was compared to the TSs of those who reported negative or positive responses regarding mistreatment for the same item.

#### 2.3.2 | Concurrent validity

The concurrent validity of the tool was evaluated through an ROC curve analysis, which involved sensitivity and specificity analysis TSs (Fawcett, 2006).

We also evaluated the degree of concordance between mistreatment classifications developed from the TS for the tool and diagnosis made by the gerontologist with the optimal cut-off point, and we explored different cut-off points for classifying mistreatment from the TS. To do this, we identified “mistreatment” when the TS > X where *x* = {1, 2, 3, 4, 5, 6, 7}. Furthermore, the concordance was evaluated using Cohen’s kappa (Higgins, 2004; Rosner, 2010). Data processing and statistical analyses were carried out using Stata 13 (StataCorp LP, 2014).

### 2.4 | Ethical considerations

The study was approved by the Commission for Ethics and Research for the National Public Health Institute of Mexico (Protocol number CI: 801, No. 696). Data were obtained, registered and processed in adherence to existing regulations on carrying out research with human beings within the country.

Written informed consent was received from the older adults who participated while oral informed consent was secured from all of the other participants. All of the informants who agreed to participate in the study did so voluntarily. We guaranteed the anonymity of all of our informants. Only the research team had access to the study data and only for the purposes of carrying out analyses and writing reports. Before apply the tool, the research team (including medical personnel) was trained. Gerontologists were on hand to provide care to participants who needed it during or after the interviews/questionnaire periods. Co-ordination with institutions caring for older adult victims of mistreatment was carried out and the team observed rules and time plans data collection set by institutions. The present study was conducted as part of the project entitled Care Models for the Mistreated Elderly [In Spanish “Modelo de Atención para Adultos Mayores Maltratados”] financed through CONACYT numbers 87671 and 248566.

## 3 | RESULTS

### 3.1 | Phase 1: Formative research

**FIGURE 1:**
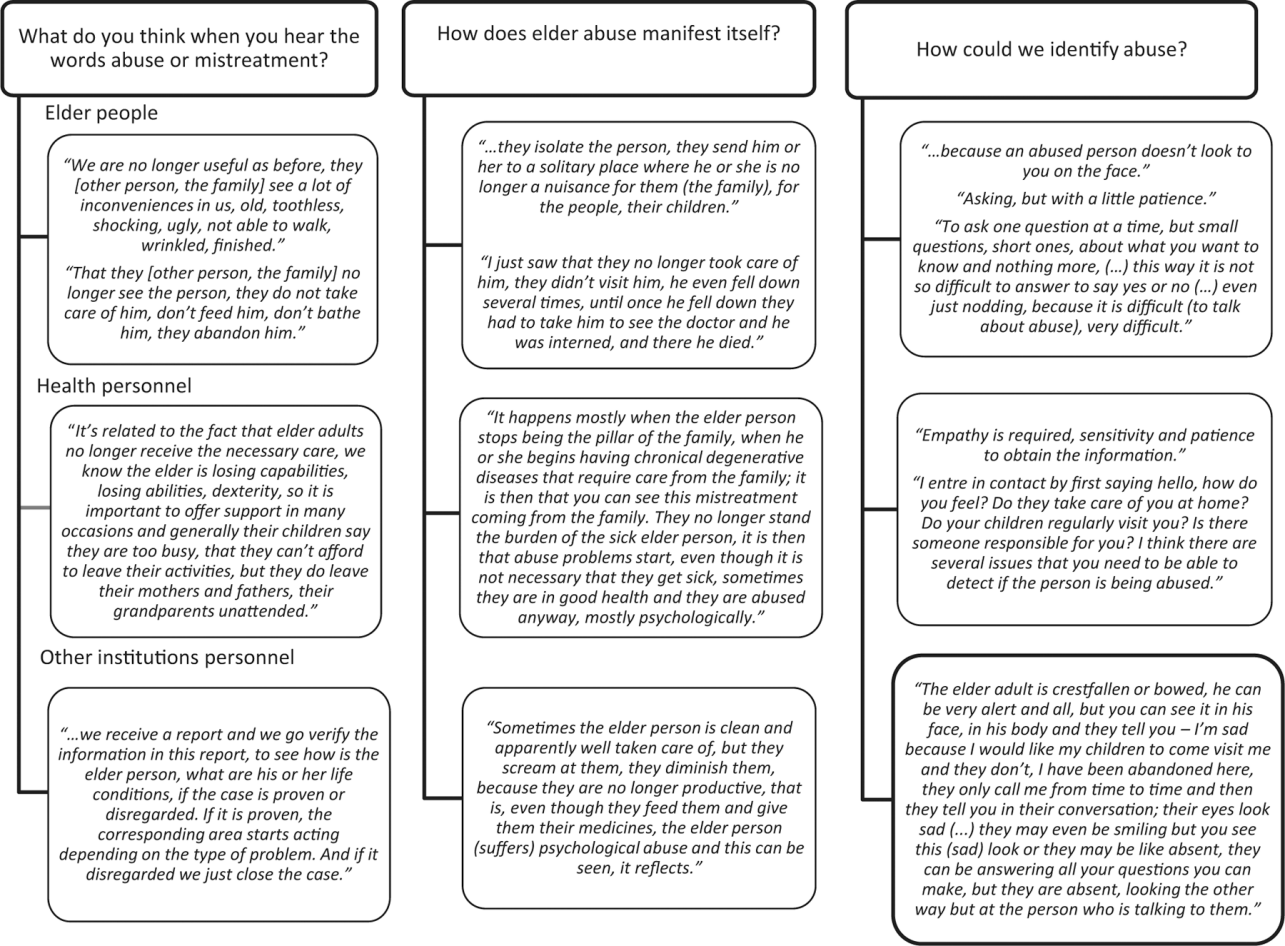
Perceptions on family mistreatment according to the different types of informants

In total, 163 semi-structured interviews were carried out with approximately 50 interviews conducted in each selected city. All of the participants were between the ages of 24 and 85, and 106 were women. Of the 63 older adults interviewed, 29 were men and 34 women, and all of them were between 60 and 85 years of age. In total, 35 of the participants reported having a partner and 59 reported having children. Only 24 had or were looking for paid employment. In addition, 40 physicians (19 women and 21 men) between the ages of 27 and 63 were interviewed. The interviewed physicians had been working in health services for between 3 months and 26 years. Thirty-four female nurses and two male nurses between the ages of 24 and 57 and with seniority of 2–24 years were interviewed. Twelve social workers and one psychology student between 23 and 51 years of age with 7 months to 21 years of experience in health services were also interviewed. A total of 12 social and legal service personnel were interviewed, and all of them were female of between 27 and 39 years of age and with 4 months to 3 years of experience. Figure 1 illustrates the informants’ perceptions regarding family mistreatment.

#### 3.1.1 | Perceptions regarding mistreatment

Older adults perceive mistreatment from their families as involving a lack of love; they identified mistreatment as an emotional matter more than physical, thus they referred to suffering as a (subjective) manifestation of that a person is being mistreated in itself. To be mistreated is to not have support from one’s family and to not be cared for, and especially when one is sick or when one needs assistance with everyday life tasks. Older adults viewed themselves as vulnerable to mistreatment from their families because when an older person gets sick or is limited in their mobility that person becomes “useless,” which in turn renders them an expensive hindrance on the family and someone who constantly requires attention. This renders an older person more vulnerable to mistreatment from family members.

For the health personnel we interviewed, the stress that families are under can be a trigger for mistreatment. Of the cases presented to legal services, psychological mistreatment, negligence and abandonment from adult children are the forms of mistreatment most frequently reported. Figure 1 presents selected quotes on the various categories of mistreatment identified, on their manifestations and on ways to identify them.

#### 3.1.2 | Manifestations of mistreatment

Our participants identified different ways in which an older adult can be mistreated: being ignored or humiliated, being criticised, being yelled at or having to witness bouts of family anger and fighting. Many of the older adults we interviewed spoke of receiving a lack of support and care while ill as another form of mistreatment. Finally, mistreatment can also result from violent behaviour that is part of a family’s dynamics.

According to the health personnel, older adults become vulnerable to mistreatment once they stop acting as pillars of their families and become “a burden” due to chronic diseases that require lifelong care. Families neglect their proper nutrition and older adults may not always receive the medical care they need. Family members may not support older adults with help and understanding in regard to everyday tasks that they can no longer carry out. For the legal personnel, mistreatment taking the form of physical aggression, yelling, humiliation and a lack of care results from the little value that society places on older adults and from a lack of recognition of their place in the family (see quotations listed in Figure 1).

#### 3.1.3 | Ways to identify mistreatment in the family

According to our participants, older adults should be asked whether they have suffered or if they feel mistreated directly, and health professionals should also explore how a mistreated older adult is feeling. According to them, asking such questions is critical as they explore how older adults feel and how they perceive and build meaning around the various expressions of mistreatment they may encounter. Older adults also stressed the need for exploration through direct follow-up questions that do not require too much explanation, as mistreated persons do not find it easy to speak about their experiences. In addition, the health and legal personnel described the need for older adults to be treated with empathy, sensitivity and patience.

In addition to the need to ask direct questions, legal service personnel highlighted the need for home visits that allow for the observation of older adult living conditions (including the evaluation of buildings themselves). Adults who are not clean and who appear malnourished should be evaluated as well. For all of the participants of our study, it was very important to be observant of the attitudes that older adults may present and of whether such attitudes suggest the presence of mistreatment. This can manifest as not being able to look anyone in the eye or as appearing frightened or distracted.

### 3.2 | Phase 2: Tool development

In total, 67 questions were created, 25, 19, 8 and 9 of which respectively addressed neglect/abandonment, psychological/emotional mistreatment, physical mistreatment, sexual mistreatment and economic mistreatment (see https://www.academia.edu/28342194/Annex_1._Questions_developed_for_the_construction_of_the_instrument_of_detection_of_abuse_in_older_adults_CALF_No). From this list, experts ranked 23 as most in line with mistreatment cases and made sure all of the selected questions could be addressed with “yes/no” answers. This first draft of the tool is shown in Annex 1.

After the first draft was completed, seven questions were modified to make them easier for older adults to understand. In total, eight questions addressed issues of psychological/emotional mistreatment; nine questions addressed issues of neglect/abandonment and two questions each addressed issues of physical, sexual and economic mistreatment. The tool was then named the Family Members Mistreatment of Older Adults Screening Questionnaire (FAMOASQ).

### 3.3 | Phase 3: Screening tool validation

#### 3.3.1 | Construct validity

A total of 253 older adults were recruited. Of these individuals, 146 presented signs of mistreatment and 107 did not. They were all given the FAMOASQ with 21 items. The congruence of each item in relation to both sample groups (the group that presented signs of mistreatment and the group that did not) is shown in Table 1. We found that the older adults presenting signs of mistreatment gave more positive responses to the 18 items with differences exceeding 20 relative to the group that did not present signs of mistreatment (*p* < .001 for 17 items with one being immeasurable). For three items (“Do they help you with your personal activities?”; “Has someone touched your body without your consent?” and “Has someone posed something to you that you consider to be improper or immoral?”), the differences fell below 11 percentage points.

Table 2 shows the results of the exploratory factor analysis. The model presents 15 questions with factor scores of above 0.4 (all with positive values considering the positive value as indicative of mistreatment). The first factor explains 83.08% of the total variance. The eigen value was measured as 5.41, while the other values fall below a value of 1. The internal consistency of the tool scored was Cronbach’s alpha = 0.89.

Median TSs for the FAMOASQ were lower for questions soliciting negative mistreatment responses compared to those for questions soliciting positive mistreatment responses with a median of 1 and interquartile range of (0, 1) and with a median of 6 and interquartile range of (3, 10) respectively. This difference was found to be statistically significant for all of the items (*p* < .0001).

#### 3.3.2 | Concurrent validity

**FIGURE 2:**
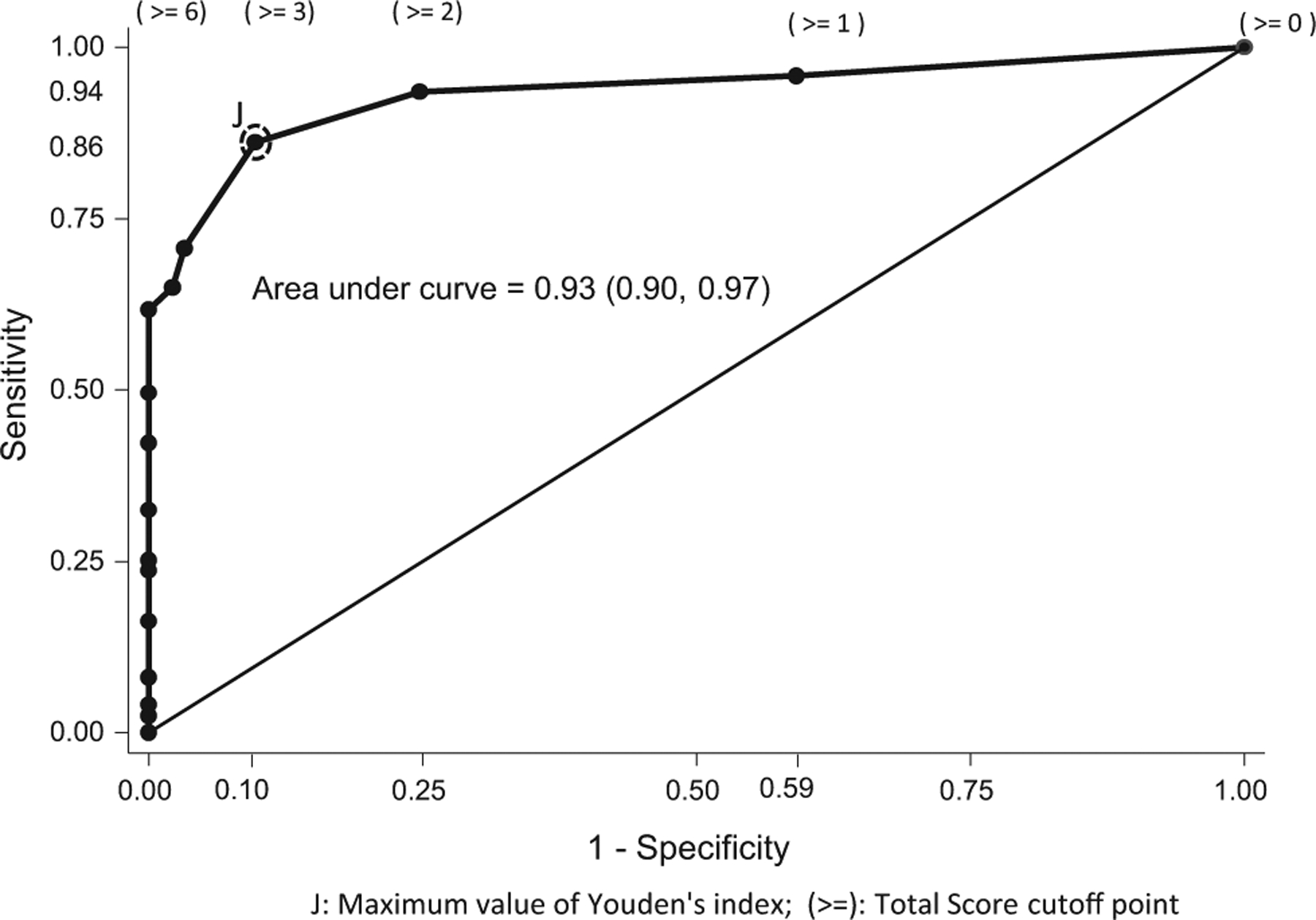
ROC analysis of Total Score for Family Members Mistreatment of Older Adults Screening Questionnaire (FAMOASQ)

Figure 2 shows the sensitivity and specificity results according to cut-off points of TS for the FAMOASQ. The optimal point is obtained when the TS cut-off point is equal to or greater than three positive items on mistreatment with a sensibility value of 86% and a specificity value of 90%. The area under curve is valued at 0.93 (0.90–0.97).

The highest degree of concordance between the FAMOASQ results and the diagnoses of mistreatment was found for the TS cut-off point of equal to or greater than three positive items (Cohen’s kappa = 0.7592, *p* < .0001). For cut-off points equal to or greater than one or two, the Cohen’s kappa values were found to be 0.4563 and 0.7089 respectively (*p* < .0001, both). For cut-off points equal to or greater than 4, 5, 6 and 7, the Cohen’s kappa values consistently decrease (Cohen’s kappa values of 0.678 to 0.5455, *p* < .0001 for all cases). Finally, the items of the FAMOASQ instrument are included in Table 3.

## 4 | DISCUSSION

The screening tool created through this study provides facilities at the first level of care and opportunities for the early identification of the familial mistreatment of older adults, which may in turn lead to adequate care and to follow-up meetings with social and legal service personnel (Perel-Levin, 2008). Its routine use at the first level of care could help reduce the deterioration of older adult health, which can in turn protect older adults from irreversible damages to their health and well-being (Bond & Butler, 2013; Burnett et al., 2014; Fulmer, Guadagno, Bitondo-Dyer, & Connolly, 2004).

The FAMOASQ is an objective and relevant tool for identifying family member mistreatment among older adults at the health services level. Not having a screening tool may cause cases of mistreatment to go unnoticed by healthcare providers either because they cannot identify it or because they have no tools to address it (Kennedy, 2005). At the second and third levels of care, mistreatment is more likely to be identified because signs and symptoms will be stronger: malnutrition, dehydration, somatisation, psychological problems, skin lesions, etc. (Eulitt, Tomberg, Cunningham, Counselman, & Palmer, 2014; Fernández-Alonso, Baratas-Crespo, García-Briñón, & Martín-Sánchez, 2011; Palmer, Brodell, & Mostow, 2013). Using this tool, health personnel can better identify cases of mistreatment and help safeguard older adults’ rights, as mistreatment is a violation of human rights that causes injuries, disease and severe psychological damage (Instituto de Mayores y Servicios Sociales, Sociedad Española de Geriatría y Gerontología, & OMS (WHO), 2007; Podnieks et al., 2010; United Nations, 2012).

The FAMOASQ is an initial quick screening tool that incorporates the perceptions of older adults with those of healthcare providers and social and legal service workers, making it different from other tools that generally only use the insights of experts (Patterson & Malley-Morrisona, 2006; Perel-Levin, 2008; WHO 2008). In addition, this is the first tool developed for the cultural, social and legal contexts of Mexico that can be administered at the first level of care.

The tool applies some contextual characteristics that other societies may share: the normalisation of mistreatment, limited perceptions of harm resulting from mistreatment, angst associated with confronting the pain of mistreatment and feelings older adults have towards their families (whether this is love or fear) that prevents such individuals from making complaints. Such complexities may help us understand why underreporting is so prevalent. It may also be that family member mistreatment of older adults is not recognised by victims and thus is not reported to healthcare providers (WHO/INPEA, 2002).

As another benefit of this tool, it does not need to be administered by an expert or highly trained healthcare provider. The tool’s items are phrased in understandable terms for older adults who speak Spanish and it can be filled out in a short amount of time. The tool can be used to identify the mistreatment of older adults who live with their families or with caregivers, as is common in many countries in Latin America (Arriagada, 2007).

The 15 items included in the FAMOASQ achieve internal and concurrent validity levels that are similar to those of tools with up to 42 items such as the “Elder Assessment Instrument” (EAI), which Burnett et al. (2014) considered to be a “good tool.” The questionnaire boasts a sensitivity level of 86% and a specificity level of 90% versus the respective 71% and 93% levels of the EAI (Burnett et al., 2014).

The final version of the tool includes more items that address issues of psychological/emotional mistreatment and neglect/abandonment in line with the comments of a panel of experts. This may be more difficult to identify than physical mistreatment (Bonnie & Wallace, 2003; Fulmer et al., 2004). However, the screening tool is useful in describing a general dimension of mistreatment, and it does not distinguish between types. This is true although it uses the WHO mistreatment typology to generate different items (Hamid et al., 2013). In addition, we compared the outcomes of the tool with expert diagnoses of cases and observed internal consistency across the 15 items based on a Cronbach’s alpha value of 0.89. The same value for the Geriatric Mistreatment Scale was measured as 0.83 with 49 items, and both are designed for use with Mexican populations (Giraldo-Rodríguez & Rosas-Carrasco, 2013).

As a limitation of this study, we were unable to blind clinical diagnoses from the team that administered the tests. This occurred due to the personal characteristics of the test takers, and especially for those identified through legal services who were unable to leave such settings for safety reasons. These interviews and clinical assessments could only be carried out in refuge areas. However, by studying individuals who had filed legal or administrative complaints related to mistreatment, we were able to secure participants who had experienced mistreatment; however, this constrained the participants’ capacities to provide their sociodemographic data to the researchers.

This study represents an initial validation of the screening tool, and so it is recommended that validations continue to ensure its reliability across the target population at the service level of care of the social security and private sectors. Using the tool at the first level of care presents several challenges to health services (e.g. developing protocols for referring older adults presenting signs of mistreatment to other health, legal and social services and providing counselling when mistreatment is first identified). The right to live a life free of violence is a human right that must be guaranteed by the Mexican state and by other countries around the world. As such, it is the state’s responsibility to set strategies and policies that guarantee the well-being of older adults in place (United Nations, 2012).

The FAMOASQ will allow for the identification of mistreatment in this population, which will in turn allow researchers and the health system to delve into an understanding of this phenomenon and to generate evidence useful for tailoring public policies to the care needs of older adults. The tool can adapt to different populations such as indigenous people in the same country. The identification and surveillance of mistreatment in older adults must become part of a health policy to guarantee the integral care of this population (United Nations, 2012). This will generate evidence that will in turn help raise awareness on this phenomenon among different actors and across society in general.

## Figures and Tables

**TABLE 1:** Analysis of the internal consistency of positive responses of the older adults to the mistreatment from their members of the family, according to the diagnosis of abuse

Items selected by the panel of experts	Response for considering mistreatment	% Elder		*p* value χ^2^
		Without mistreatment (*n* = 107)	With mistreatment (*n* = 146)	
Do they help you with your personal activities? (to take a bath or to dress, to eat or to go shopping or to the bank)	No	20	31.5	.043
Do they help you with your medication?	No	17.7	57.1	<.001
Do you feel you are the cause of problems? (some kind of burden)	Yes	14.3	46.2	<.001
Is your family usually angry at you? (all the time, regularly)	Yes	12.4	38.5	<.001
Do they take you to see a doctor?	No	10.9	39.3	<.001
Do you go out for pleasure or entertainment with your family?	No	10.5	54.6	<.001
Have you been emotionally hurt?	Yes	9.5	58.6	<.001
Does your family visit you? (frequently, all the time)	No	8.6	52.1	<.001
Have you been hurt doing work at home? (Cooking, sweeping, mopping, use tools, caring for others)	Yes	8.6	27.9	<.001
Have you been left alone for long periods of time? (a long time)	Yes	5.7	49.6	<.001
Does the person you live with ignore you? (Does not pay attention to you, does not consider you)	Yes	4.8	53.6	<.001
Has someone taken your money or belongings without your consent? (without telling you, without your permission)	Yes	4.8	27	<.001
When you ask your family for help, do they help you?	No	3.9	40	<.001
Do you feel abandoned?	Yes	3.8	44.8	<.001
When you are sick or do not feel well, does someone accompany you?	No	2.9	35	<.001
Are you always afraid of something?	Yes	2.9	34.3	<.001
Do you handle your own money?	No	2.9	23.6	<.001
Do you feel you are not respected?	Yes	1.9	50	<.001
Do you trust the person you live with?	No	1	30	<.001
Has someone beaten or hurt you physically?	Yes	1	15.3	<.001
Do you feel you are threatened?	Yes	0	26.6	-
Has someone touched your body without your consent? (fumbled you)	Yes	0	4.2	-
Has someone proposed to you something that you consider improper or immoral? (nasty, dirty, daring, indecent)	Yes	0	2	-

**TABLE 2:** Exploratory factor analysis of Family Members Mistreatment of Older Adults Screening Questionnaire to identify domestic abuse in elderly

Item number first set	Items	Factor 1	Factor 2	Factor 3	Factor 4	Factor 5	Factor 6	Factor 7	Factor 8
	Eigen value	5.4156	0.9855	0.4356	0.2867	0.1998	0.1552	0.0756	0.0006
43	Do you feel abandoned?	0.713	−0.023	−0.150	−0.142	−0.127	0.164	0.013	0.000
6	Does the person you live with ignore you? (Does not pay attention to you, does not consider you)	0.663	−0.141	0.159	0.058	−0.096	−0.091	−0.091	−0.003
7	Have you been emotionally hurt?	0.661	−0.152	0.259	−0.108	0.038	−0.121	0.070	−0.006
1	Do you feel you are not respected?	0.644	−0.380	0.068	−0.110	−0.078	−0.078	−0.059	0.006
8	Do you feel you are threatened?	0.623	−0.333	−0.315	0.033	0.139	−0.039	0.024	0.001
51	Does your family visit you? (frequently, all the time)	0.612	0.193	−0.049	−0.183	−0.129	−0.075	0.117	−0.005
3	Are you always afraid of something?	0.611	−0.321	−0.112	0.049	0.222	0.010	−0.029	−0.007
50	Do they help you with your personal activities? (to take a bath or to dress, to eat or to go shopping or to the bank)	0.610	0.175	−0.038	−0.242	0.046	0.011	−0.038	0.011
46	When you are sick or do not feel well, does someone accompany you?	0.609	0.407	0.125	−0.039	0.182	0.070	−0.036	0.001
4	Do you trust the person you live with?	0.581	0.178	0.103	0.041	−0.054	0.116	−0.103	−0.013
44	Do you go out for pleasure or entertainment with your family?	0.580	0.164	−0.026	0.267	−0.107	−0.154	−0.037	0.007
45	Have you been left alone for long periods of time? (a long time)	0.550	0.325	−0.346	0.092	−0.064	−0.018	−0.029	−0.002
2	Is your family usually angry at you? (all the time, regularly)	0.506	−0.195	0.191	0.123	−0.021	0.185	0.004	0.010
5	Do you feel you are the cause of problems? (some kind of burden)	0.506	−0.142	0.024	0.188	−0.075	0.108	0.148	−0.001
48	Do they help you with your medication?	0.498	0.358	0.132	0.103	0.149	−0.058	0.079	0.003

**TABLE 3:** Family Members Mistreatment of Older Adults Screening Questionnaire (FAMOASQ).

Recommendations:		
The interviewer must be objective Establish a confidence relationship with the elder person To do the interview privately, in empathic surroundings that guarantee confidentiality Words in parenthesis may be used to improve the understanding of the questions		
I am now going to ask you a series of questions related to the actions that may be present in **your family**. It is important that you consider the way you perceive each situation.		
Num.	Question	Answer to consider mistreatment
1	Do you feel abandoned?	Yes
2	Have you been left alone for long periods of time? (a long time)	Yes
3	Does your family visit you? (frequently, all the time)	No
4	Do they help you with your personal activities? (to go shopping or to the bank)	No
5	When you are sick or do not feel well, does someone accompany you?	No
6	Do they help you with your medication?	No
7	Do you trust the person you live with?	No
8	Do you go out for pleasure or entertainment with your family?	No
9	Is your family usually angry at you? (all the time, regularly)	Yes
10	Have you been emotionally hurt?	Yes
11	Are you always afraid of something?	Yes
12	Do you feel you are threatened?	Yes
13	Do you feel you are the cause of problems? (some kind of burden)	Yes
14	Do you feel you are not respected?	Yes
15	Does the person you live with ignore you? (Does not pay attention to you, does not consider you)	Yes

Source: Ruelas González y cols. Research Project “Modelo de Atención para adultos mayores maltratados”, funded by CONACYT 87671 and 248566. Interpretation of evaluation to more than three positive responses to mistreatment considers the possibility of being an older adult with family mistreatment.

## What is known about this topic

- Family mistreatment is a medical and social problem for ageing populations. However, it tends to be under-reported in healthcare facilities.
- Mistreatment of older adults is usually not detected in health services.
- Screening tools to identify the mistreatment of older adults are frequently developed for experts and tend to not be understood by older adults themselves.

## What this paper adds

- A screening tool integrating perceptions from older adults and those of the health, social and legal personnel appointed to provide care and services.
- A tool that screens for mistreatment of older adults by their families, with high sensitivity and specificity.
- A tool with high internal consistency for a total scale.

## References

[R1] Acierno R, Hernandez-Tejada M, Muzzy W, & Steve K (2009). Final report: National elder mistreatment study. Retrieved from https://www.ncjrs.gov.

[R2] Adams YC (2012). Maltrato familiar en el adulto mayor institucionalizado realidad e invisibilidad [Domestic abuse in the elderly institutionalized reality and invisibility]. Revista Médica Clínica Las Condes, 23, 84–90.

[R3] Almogue A, Weiss A, Marcus EL, & Beloosesky Y (2009). Attitudes and knowledge of medical and nursing staff toward elder abuse. Archives of Gerontology and Geriatrics, 51, 86–91. 19775762 10.1016/j.archger.2009.08.005

[R4] Aravanis SC, Adelman RD, Breckman R, Fulmer T, Holder E, Lachs MS, … Sanders AB (1993). Diagnostic and treatment guidelines on elder abuse and neglect. Archives of Family Medicine, 2, 371–388. 8130916 10.1001/archfami.2.4.371

[R5] Arriagada I (2007). Familias y Políticas Públicas en América Latina. Una historia de desencuentros. [Families and Public Policy in Latin America. A history of disagreements]. de la CEPAL Libros, 96. Santiago de Chile: Comisión Económica para América Latina y el Caribe (CEPAL). Retrieved from http://repositorio.cepal.org/bitstream/handle/11362/2504/S0700488_es.pdf?sequence=1.

[R6] Barraza-Macías A (2007). La consulta a expertos como estrategia para la recolección de evidencias de validez basadas en el contenido Apuntes sobre metodología de la investigación [Expert consultation as a strategy for collecting evidence of validity based on content. Notes on research methodology]. Universidad Pedagógica de Durango, 7, 514.

[R7] Bond MC, & Butler KH (2013). Elder abuse and neglect. Definitions, epidemiology and approaches to emergency department screening. Clinics in Geriatric Medicine, 29, 257–273. 23177610 10.1016/j.cger.2012.09.004

[R8] Bonnie RJ, & Wallace RB (2003). Screening and case identification in clinical settings. Elder mistreatment: Abuse, neglect, and exploitation in an aging America. The National Academies Press. Retrieved from http://www.ncbi.nlm.nih.gov/books/NBK98788/. 22812026

[R9] Burnett J, Achenbaum WA, & Murphy KP (2014). Prevention and early identification of elder abuse. Clinics in Geriatric Medicine, 30, 743–759. 25439639 10.1016/j.cger.2014.08.013

[R10] Consejo Nacional de Población. (2011). Índices de marginación por entidad federativa y municipio 2010. [Marginalization by state and municipality 2010 indexes]. Retrieved from http://www.conapo.gobmx/es/CONAPO/Indices_de_Marginacion_2010_por_entidad_federativa_y_municipio.

[R11] Cooper C, Selwood A, & Livingston G (2008). The prevalence of elder abuse and neglect: A systematic review. Age and Ageing, 37, 151–160. 18349012 10.1093/ageing/afm194

[R12] Cresswell JW (2007). Qualitative inquiry and research design: Choosing among five Approaches. London: Sage.

[R13] Echeverría S, Sotelo M, Barrera L, & López M (2013). Diseño de instrumentos de medición en Psicología y sus propiedades psicométricas: Competencia metodológica en estudios de Psicología [Design of measurement instruments in Psychology and its psychometric properties: Methodological competence in Psychology studies]. Cd. Obregón: Instituto tecnológico de Sonora.

[R14] Eulitt PJ, Tomberg RJ, Cunningham TD, Counselman FL, & Palmer RM (2014). Screening elders in the emergency department at risk for mistreatment: A pilot study. Journal of Elder Abuse and Neglect, 26, 424–435. 24635639 10.1080/08946566.2014.903549

[R15] Fawcett T (2006). An introduction to ROC analysis. Pattern Recognition Letters, 27, 861–874.

[R16] Fernández-Alonso C, Baratas-Crespo E, García-Briñón MÁ, & Martín-Sánchez FJ (2011). Deteccción de malos tratos al anciano en las urgencias hospitalarias [Detection of abuse of the elderly in hospital emergency departments]. Atención Primaria, 43, 451–452. 21295885 10.1016/j.aprim.2010.05.020 PMC7024936

[R17] Flick U (2007). Qualitative research designs. Designing qualitative research. London: Sage. Retrieved from 10.4135/9781849208826.

[R18] Folstein MF, Folstein SE, & McHugh PR (1975). “Mini-mental state”. A practical method for grading the cognitive state of patients for the clinician. Journal of Psychiatric Research, 12, 189–198. 1202204 10.1016/0022-3956(75)90026-6

[R19] Fulmer T, Guadagno L, Bitondo-Dyer C, & Connolly MT (2004). Progress in elder abuse screening and assessment instruments. Journal of the American Geriatrics Society, 52, 297–304. 14728644 10.1111/j.1532-5415.2004.52074.x

[R20] Giraldo-Rodríguez L, & Rosas-Carrasco O (2013). Development and psychometric properties of the Geriatric Mistreatment Scale. Geriatrics and Gerontology International, 13, 466–474. 22694594 10.1111/j.1447-0594.2012.00894.x

[R21] Hair JF, Black WC, Babin BJ, & Anderson RE (2014). Multivariate data analysis (7th edn). Harlow, United Kingdom: Pearson.

[R22] Hamid TA, Momtaz YA, Ibrahim R, Mansor M, Samah AA, Yahaya N, & Abdullah SFZ (2013). Development and psychometric properties of the Malaysian elder abuse scale. Open Journal of Psychiatry, 3, 283–289.

[R23] Higgins JJ (2004). Introduction to modern nonparametric statistics (4th edn). Pacific Grove, California: Brooks/Cole-Thomson Learning.

[R24] Holland PW, & Wainer H (2009). Differential item functioning. London: Routledge.

[R25] Huenchuán S, & Rodríguez-Piñero L (2014). Envejecimiento y derechos humanos: situación y perspectivas de protección [Aging and human rights: Current situation and perspectives of protection]. Santiago de Chile: CEPAL/UNFPA/Asdi.

[R26] Hwalek MA, & Sengstock MC (1985). A screening instrument for identifying elderly at risk of abuse and neglect. Paper presented at the 38th Annual Scientific Meeting of the Gerontological Society of America, New Orleans. Retrieved from http://files.eric.ed.gov/fulltext/ED264490.pdf.

[R27] Hwalek MA, Sengstock MC, & Lawrence R (1984). Assessing the probability of abuse of the elderly. San Antonio, TX: Paper presented at the Annual Scientific Meeting of the Gerontological Society (38th, San Antonio, TX, November 16–20). Administration on Aging (DHHS), Retrieved from http://eric.ed.gov/?id=ED257016

[R28] Instituto de Mayores y Servicios Sociales, Sociedad Española de Geriatría y Gerontología, & OMS (WHO). (2007). Malos Tratos a Personas Mayores. Aportación Española a los avances internacionales en la adaptación lingüística y cultural de un instrumento de detección de sospecha de maltrato familiar hacia las personas mayores. [Mistreatment of Elderly. A Spanish contribution to international developments in the linguistic and cultural adaptation of an instrument of detection of suspected domestic abuse towards the elderly]. Colección Documentos. Serie Documentos Técnicos No. 21013. Madrid: Ministerios de Trabajo y Asuntos Sociales/ IMSERSO.

[R29] Kennedy RD (2005). Elder abuse and neglect: The experience, knowledge, and attitudes of primary care physicians. Family Medicine-Kansas City, 37, 481. 15988632

[R30] Kosberg JI, Lowenstein A, Garcia JL, & Biggs S (2003). Study of elder abuse within diverse cultures. Journal of Elder Abuse and Neglect, 15, 7189.

[R31] Krug EG, Dahlberg LL, Mercy JA, Zwi AB, & Lozano R (2002). World report on violence and health. Geneva: World Health Organization. Retrieved from http://whqlibdoc.who.int/hq/2002/9241545615_eng.pdf.

[R32] Lindenbach JM, Larocque S, Lavoie AM, & Garceau ML (2012). Older adult mistreatment risk screening: Contribution to the validation of a screening tool in a domestic setting. Canadian Journal on Aging/La Revue Canadienne du Vieillissement, 31, 235–252. 10.1017/S0714980812000153 22647665

[R33] LoFaso VM, & Rosen T (2014). Medical and laboratory indicators of elder abuse and neglect. Clinics in Geriatric Medicine, 30, 713–728. 25439637 10.1016/j.cger.2014.08.003

[R34] Martínez J, & Brenes Y (2007). Maltrato familiar, negligencia y abandono de la persona adulta mayor costarricense. Costa Rica: Caja costarricense de seguro social/Hospital Nacional de Geriatría y Gerontologia/Trabajo social.

[R35] Palmer M, Brodell RT, & Mostow E (2013). Elder abuse: Dermatologic clues and critical solutions. Journal of the American Academy of Dermatology, 68, e3742. 10.1016/j.jaad.2011.03.016 23058735

[R36] Patterson M, & Malley-Morrisona K (2006). A cognitive ecological approach to elder abuse in five cultures: Human rights and education. Educational Gerontology, 32, 7382.

[R37] Perel-Levin S (2008). Discussing screening for elder abuse at primary health care level. Geneva: World Health Organization.

[R38] Podnieks E, Penhale B, Goergen T, Biggs S, & Han D (2010). Elder mistreatment: An international narrative. Journal of Elder Abuse and Neglect, 22, 131–163. 20390829 10.1080/08946560903436403

[R39] Quero-Virla M (2010). Confiabilidad y coeficiente Alpha de Cronbach. TELOS. Revista de Estudios Interdisciplinarios en Ciencias Sociales, 12, 248–252.

[R40] Rinaldi-Carpenter D (2011). Phenomenology as method. In Streubert HJ, & Rinaldi-Carpenter D (Eds.), Qualitative Research in Nursing: Advancing the humanistic imperative (pp. 72–95). Philadelphia: Wolters Kluwer/ Lippincott Williams & Wilkins.

[R41] Rosner B (2010). Fundamentals of biostatistics. Kentucky: Cengage Learning. Inc.

[R42] Ruelas-González MG, Duarte-Gómez MB, Flores-Hernández S, Ortega-Altamirano DV, Cortés-Gil JD, Taboada A, & Ruano AL (2016). Prevalence and factors associated with violence and abuse of older adults in Mexico′s National Health and Nutrition Survey. International Journal of Equity in Health, 15, 35. 10.1186/s12939-016-0315-y PMC4769586 26920364

[R43] Ruelas-González MG, Pelcastre-Villafuerte BE, & Reyes-Morales H (2014). Maltrato institucional hacia el adulto mayor: Percepciones del prestador de servicios de salud y de los ancianos. Salud Publica Mexico, 56, 631–637. 25604415

[R44] Santos JRA (1999). Cronbach’s alpha: A tool for assessing the reliability of scales. The Journal of Extention, 37(2), 1–5.

[R45] Secretaría de Salud. (2009). Guía de Práctica Clínica para Detección y Manejo del Maltrato familiar en las personas adultas mayores en el Primer Nivel de Atención. [Clinical practice guideline for detection and management of family abuse in elders at the primary care level].

[R46] Sellas MI (2004). Elder abuse. Department of Emergency Medicine, Harvard Medical School, Brigham and Women’s Hospital/Massachusetts General Hospital Harvard Medical School.

[R47] StataCorp LP. (2014). Stata 13. College Station: StataCorp LP.

[R48] United Nations. (2012). Resolution adopted by the General Assembly on 19 December 2011 (9 march 2012). A/RES/66/127.

[R49] WHO. (2002). The Toronto declaration on the global prevention of elder abuse. Retrieved from www.who.int.

[R50] WHO. (2008). A global response to elder abuse and neglect: Building primary health care capacity to deal with the problem worldwide: Main report. Retrieved from www.who.int/ageing/publications/ELDER_DocAugust08.pdf.

[R51] WHO. (2015). World report on ageing and health. Retrieved from www.who.int/entity/ageing/publications/world-report-2015/en/.

[R52] WHO and International Network for the Prevention of Elder Abuse, WHO/INPEA. (2002). Missing voices: Views of older persons on elder abuse. Geneva: World Health Organization. Retrieved from www.who.int

[R53] Williams B, Onsman A, & Brown T (2010). Exploratory factor analysis: A five step guide for novices. Journal of Emergency Primary Health Care, 8(3). Retrieved from https://ajp.paramedics.org/index.php/ajp/article/view/93/90.

